# Increased Diurnal Activity Is Indicative of Energy Deficit in a Nocturnal Mammal, the Aardvark

**DOI:** 10.3389/fphys.2020.00637

**Published:** 2020-07-07

**Authors:** Nora Marie Weyer, Andrea Fuller, Anna Jean Haw, Leith Carl Rodney Meyer, Duncan Mitchell, Mike Picker, Benjamin Rey, Robyn Sheila Hetem

**Affiliations:** ^1^Brain Function Research Group, School of Physiology, Faculty of Health Sciences, University of the Witwatersrand, Johannesburg, South Africa; ^2^Centre for Veterinary Wildlife Studies and Department of Paraclinical Sciences, Faculty of Veterinary Science, University of Pretoria, Pretoria, South Africa; ^3^Research Institute of Wildlife Ecology, Department of Integrative Biology and Evolution, University of Veterinary Medicine Vienna, Vienna, Austria; ^4^Department of Biological Sciences, Faculty of Science, University of Cape Town, Cape Town, South Africa; ^5^Laboratoire de Biométrie et Biologie Evolutive, UMR 5558, Université de Lyon, Université Lyon 1, CNRS, Villeurbanne, France; ^6^School of Animal, Plant and Environmental Sciences, Faculty of Science, University of the Witwatersrand, Johannesburg, South Africa

**Keywords:** body temperature, heterothermy, biologging, behavioural flexibility, climate change

## Abstract

Shifting activity to cooler times of day buffers animals from increased heat and aridity under climate change. Conversely, when resources are limited, some nocturnal species become more diurnal, reducing energetic costs of keeping warm at night. Aardvarks (*Orycteropus afer*) are nocturnal, obligate ant- and termite-eating mammals which may be threatened directly by increasing heat and aridity, or indirectly by the effects of climate change on their prey. We hypothesised that the minimum 24-h body temperature of aardvarks would decline during energy scarcity, and that aardvarks would extend their active phases to compensate for reduced resource availability, possibly resulting in increased diurnal activity when aardvarks were energetically compromised. To measure their thermoregulatory patterns and foraging activity, we implanted abdominal temperature and activity data loggers into 12 adult aardvarks and observed them for varying durations over 3 years in the Kalahari. Under non-drought conditions, aardvarks tightly controlled their 24-h body temperature rhythm (mean amplitude of the 24-h body temperature rhythm was 1.8 ± 0.3°C during summer and 2.1 ± 0.1°C during winter) and usually were nocturnal. During a summer drought, aardvarks relaxed the precision of body temperature regulation (mean 24-h amplitude 2.3 ± 0.4°C) and those that subsequently died shifted their activity to progressively earlier times of day in the weeks before their deaths. Throughout the subsequent winter, the aardvarks’ minimum 24-h body temperatures declined, causing exaggerated heterothermy (4.7 ± 1.3°C; absolute range 24.7 to 38.8°C), with one individual’s body temperature varying by 11.7°C within 8 h. When body temperatures were low, aardvarks often emerged from burrows during daytime, and occasionally returned before sunset, resulting in completely diurnal activity. Aardvarks also shortened their active periods by 25% during food scarcity, likely to avoid energetic costs incurred by foraging. Despite their physiological and behavioural flexibility, aardvarks were unable to compensate for reduced food availability. Seven study aardvarks and several others died, presumably from starvation. Our results do not bode well for aardvarks facing climate change, and for the many animal species dependent on aardvark burrows for refuge.

## Introduction

Large mammals that reproduce slowly and are unable to move to more suitable surroundings require flexibility in behaviour and physiology to survive in rapidly changing environments (Fuller et al., 2016). Understanding flexibility in activity patterns of mammals is crucial for predicting their resilience to climate change (McCain and King, 2014). Many mammals buffer the effects of increasing heat and aridity, occurring with climate change, by seeking cooler microclimates and reducing diurnal activity (Hetem et al., 2012a; McFarland et al., 2014). Those adjustments reduce the demand for water for evaporative cooling. If cooler microclimates are not available or if reduced diurnal activity compromises energy intake, diurnal species may increase their nocturnal activity (Hetem et al., 2012a; Levy et al., 2019).

Nocturnal species already are active at the cooler time of day, so one might predict less change in their activity patterns in response to increasing heat and aridity. By contrast with diurnally active mammals that might need to increase evaporative water loss to keep cool, mammals active during the night might need to increase their metabolic rates to keep warm, which may impose an extra demand for energy during cold nights. Indeed, increasing air temperatures at night, as a result of climate change, might benefit nocturnal species by reducing the energetic costs of maintaining homeothermy (Levy et al., 2019), a benefit that might help offset the reduced nutritional quality or availability of food resulting from climate change (Durant et al., 2007; Post et al., 2008; Birnie-Gauvin et al., 2017). In environments with clear night skies, substantial heat loss occurs through passive radiant cooling to the night sky, which acts as a heat sink with an effective temperature of between –10 and –30°C (Mitchell et al., 2018). Because the radiant heat sink of the night sky is unlikely to change much with climate change, warmer air temperatures at night are unlikely to offset the energetic cost of reduced resource availability. Instead, as resources become limited, the ability to keep warm at night using metabolic heat might be compromised, such that some nocturnal species may increase their diurnal activity to reduce the energy cost that would have been incurred through being active at night (Van der Vinne et al., 2019). The trade-off then is that they have to acquire sufficient resources while exposed to daytime heat loads, with air temperatures increasing under climate change.

Alterations in activity patterns across the day, as well as changes in energy or water balance, have implications for body temperature regulation. Periods of energy deficit resulting from reduced food availability or increased energy expenditure are associated with low minimum 24-h body core temperatures (Fuller et al., 2014; Hetem et al., 2016) for many large mammal species (Maloney et al., 2011; Signer et al., 2011; Brinkmann et al., 2012; Hetem et al., 2012b; Lubbe et al., 2014; Whiteman et al., 2015). On the other hand, large mammals exposed to water deficit in hot and dry environments exhibit high maximum 24-h body temperatures (Mitchell et al., 2002; Hetem et al., 2010; Fuller et al., 2014), thereby reducing water loss through evaporative cooling. Such increases in the maximum 24-h and decreases in the minimum 24-h body temperatures result in increases in the amplitude of the 24-h body temperature rhythm. Because an increased amplitude of the 24-h body temperature rhythm, termed heterothermy, is associated with an energy deficit or water deficit or both, it reflects a disruption of the physiological well-being of large mammals in their natural environment (Fuller et al., 2014). Indeed, homeothermy appears to occur only in healthy, undisturbed large mammals with sufficient access to food and water (Hetem et al., 2016).

One species that exhibits increased heterothermy in the face of heat and aridity is the aardvark, *Orycteropus afer* (Rey et al., 2017). Aardvarks are solitary, burrowing mammals that occur in non-desert habitats of sub-Saharan Africa. They extract ants and termites, on which they depend for energy and water, by digging them out of their underground hives (Taylor, 1998; Weyer, 2018). Aardvarks generally emerge from their burrows at night to forage, but demonstrate flexibility in behaviour and activity patterns in response to environmental fluctuations. Aardvarks in the semi-arid Karoo region of South Africa displayed an increased amplitude of their 24-h body temperature rhythm during winter when it was cold, as a result of low minimum 24-h body temperatures (Taylor and Skinner, 2004), whereas aardvarks in the more-arid Kalahari region of South Africa displayed an increased amplitude of the 24-h body temperature rhythm during a summer drought, as a result of low minimum 24-h body temperatures (Rey et al., 2017). In both studies, the low body temperatures were accompanied by an increase in diurnal activity. In the Kalahari, many aardvarks (including five out of six study aardvarks) died during the drought, presumably from starvation, and survivors were in poor condition (Rey et al., 2017). It remains unclear whether the shift to diurnal activity resulted from aardvarks being intolerant of the cold night-time temperatures in winter (Taylor and Skinner, 2004), or from an attempt to reduce the energetic costs of homeothermy when resources were limited (Rey et al., 2017). Here, we expand our previous study to investigate the key drivers of changes in body temperature and activity in aardvarks.

We assessed the consequences of fluctuating resource availability and environmental variables on body temperature rhythms and activity patterns of aardvarks in the Kalahari. We hypothesised that minimum 24-h body temperature of aardvarks would decline during periods of low food resource availability, resulting in an increased amplitude of the 24-h body temperature rhythm. Secondly, we hypothesised that to compensate for reduced food availability or increased energetic cost to maintain homeothermy during cooler times of the year (in winter), aardvarks might become increasingly diurnal when they were more compromised energetically.

## Materials and Methods

### Study Site

The study was carried out between July 2012 and September 2015 at Tswalu, a private nature reserve in the Northern Cape Province of South Africa (S 27°14′, E 22°22′). The dominant vegetation type comprised Kalahari thornveld (Acocks, 1988) and shrubby Kalahari dune bushveld on the plains (Low and Rebelo, 1998). The aardvark study site was dominated by Gordonia duneveld vegetation (Tokura, 2016). Large carnivores present were leopards (*Panthera pardus*) and cheetahs (*Acinonyx jubatus*). African wild dogs (*Lycaon pictus*) were introduced in 2014. Tswalu is situated within an area that is predicted to become hotter and drier under future climate change (Engelbrecht et al., 2015), and already experiences low and highly variable rainfall, including sporadic droughts (Thomas et al., 2007). The majority of rain falls between December and April (Davis et al., 2010; Tokura, 2016). The hot season in which air temperatures peak at ∼35°C lasts from November to February, which overlaps with the period during which rainfall typically occurs. The cold season in which air temperatures frequently drop below zero lasts from June to September and typically coincides with the driest period.

### Climatic Variables

We recorded the air temperature at the study site at 30-min intervals using a weather station (Watchdog 2700, Spectrum Technologies Inc., United States; data provided by A. Young, Exeter University). From these data, we calculated for each 24-h period the minimum, maximum, and mean air temperatures. We assessed local long-term rainfall over the 33 years, including 30 years preceding our study (1982–2015; each year starting in July and ending the following June) using FLDAS_NOAH010 data provided via the online data system Giovanni, the Goddard Earth Sciences Data and Information Services Center (GES DISC) Interactive Online Visualization and Analysis Infrastructure, developed and maintained by the NASA GES DISC^1^ (Acker and Leptoukh, 2007). During our study, we recorded rainfall on the study site using an event logger (HOBO Pendant Event data logger, Onset Computer Corporation, United States) and tipping bucket (Davis Instruments Corporation, United States). To assess rainfall during the growing season before our study started, we obtained rainfall data from the rainfall database of Tswalu. The two datasets showed high levels of agreement during overlapping measurement periods. We calculated total rainfall per month for the period January 2012 to December 2015. For the same period, we obtained daily times of sunrise and sunset for the nearest town (Upington, situated 200 km southwest of the study site^2^), from which we calculated the daily photoperiod.

### Aardvark Capture and Surgery

Over a 3.5 year period, 12 individual aardvarks were darted and implanted with data loggers (see below) to record their body temperature and activity. At different timepoints throughout the study, our sample size ranged between one and five individuals for body temperature and one and four individuals for activity, as a result of aardvark deaths and failure of biologging devices (see Supplementary Table S1 for details). In July 2012, we instrumented six aardvarks; five of these aardvarks died in March 2013 during the summer drought (Rey et al., 2017) and we retrieved biologgers from four of their carcasses (*A01*, *A02*, *A04*, and *A05*). In July 2013, we recaptured the surviving aardvark (*A06*), and instrumented six additional aardvarks (aardvarks *A07*–*A12*). One aardvark (*A10*) died during the winter of 2013. One aardvark (*A11*) died in the early summer of 2014, nearly 17 months after implantation, with no prior signs of body condition deterioration or weakness. In July 2014, we removed and reimplanted biologgers into two aardvarks (*A07* and *A08*), and one aardvark (*A12*) was added to the study in July 2014 for the third study year (July 2014 to September 2015; see Supplementary Table S1 for details). Another died in winter 2015 (*A09*), after its body condition deteriorated. Aardvark body mass during capture averaged 35 kg (range 26.5–43.0 kg). Determination of sex was not done for the aardvarks captured before 2013. All aardvarks captured after 2013 (*A06*–*A12*) were females, as classified based on a published description of aardvark genitalia (Pocock, 1924).

We captured and immobilised our aardvarks as previously described (Rey et al., 2014). Each aardvark was immobilised using a propelled dart administered intramuscularly. Each dart was filled with a combination of 150 mg ketamine, 4 mg medetomidine, and 10 mg midazolam using concentrated drugs (ketamine: 200 mg mL^–1^; medetomidine: 50 mg mL^–1^; and midazolam: 50 mg mL^–1^; Kyron Laboratories, South Africa), and topped up with sterile water to reach a final volume of 2 mL. Anaesthesia was maintained by inhalation of isoflurane (Isofor, 0.5–6%, SafeLine Pharmaceuticals, South Africa) in 100% medical oxygen. During surgery, each aardvark received a ringer’s lactate drip administered at a maintenance rate of 5 mL kg^–1^ h^–1^. We monitored respiration (respiratory rate, peripheral haemoglobin oxygen saturation, and end-tidal carbon dioxide) and cardiovascular (pulse rate and arterial blood pressure) variables, and rectal temperature throughout anaesthesia. A ∼100 × 100 mm section of the paralumbar region of each aardvark was shaved, washed and sterilised using antiseptic solutions (chlorhexidine: 5%, chlorhexidine gluconate: 0.5% in alcohol, F10 Products, Health and Hygiene, South Africa). Local anaesthetic (lignocaine hydrochloride: 2%, 3 mL, Bayer Animal Health, South Africa) was injected subcutaneously at the surgical site. Additionally, each aardvark received a long-acting antibiotic intramuscularly (Duplocillin, procaine benzylpenicillin: 6,000 IU kg^–1^, Intervet, South Africa), a non-steroidal anti-inflammatory (meloxicam: 0.5 mg kg^–1^, Boehringer Ingelheim Pharmaceuticals, South Africa) and an opioid analgesic (buprenorphine hydrochloride: 0.01 mg kg^–1^, Reckitt Beckinser Healthcare, United Kingdom) subcutaneously.

A 30–50 mm incision was made through the skin, muscle layers, and parietal peritoneum, through which the temperature data logger and very high frequency (VHF) transmitter (see below) were inserted into the abdominal cavity. The data logger that recorded locomotor activity of the aardvark was inserted and tethered intramuscularly (*Musculus transversus abdominis*) using non-absorbable polyamide suture (Nylon, Scimitar Surgical Sutures, Gabler Medical, United Kingdom), to ensure that the activity records were not affected by free movement of the logger inside the animal’s body. Incisions were closed using absorbable polyglycolic acid suture material (Viamac, Scimitar Surgical Sutures, Gabler Medical, United Kingdom). The wound was sprayed with a topical antiseptic (Necrospray, oxytetracycline hydrochloride: 40 mg, gentian violet: 4 mg, Animal Health Division − Bayer HealthCare, South Africa) and coated with a topical ectoparasiticide (Tick Grease, chlorfenvinphos: 0.3%, SWAVET RSA, South Africa). After surgery, each study aardvark was released as close as possible to its capture site, where a drug to reverse the immobilisation was administered slowly intravenously (Antisedan, atipamezole hydrochloride: 0.5 mg kg^–1^, Pfizer Laboratories, South Africa). For further details about surgical procedures and data logger implantation, see Weyer (2018).

We closely observed recovery of each aardvark during the weeks following surgery. Each study aardvark was recaptured annually, and a similar procedure to that used for implants was followed to replace, or remove, the loggers and tracking transmitter. The implant sites had healed and no signs of infection from the implantation surgery were visible at recapture.

### Data Loggers and Tracking Transmitter

We implanted two data loggers and one tracking transmitter into each aardvark (total mass of implanted equipment ∼300 g, <1% of aardvark body mass; see Supplementary Table S2 for dimensions of all implanted devices). Transmitters and data loggers were covered with inert wax (Sasolwax 1276, Sasol Ltd., South Africa) and sterilised in formaldehyde vapour before implantation. The tracking transmitter (Africa Wildlife Tracking, South Africa) allowed relocation of aardvarks in the field with a directional antenna and receiver kit (Three-Element Folding Yagi Antenna, R1000 Receiver, Sirtrack, New Zealand). A temperature-sensitive data logger recorded abdominal body temperature in each aardvark (2012–2013: DS1922L Thermochron iButton^®^, Dallas Semiconductor/Maxim Integrated Products, United States; resolution 0.0625°C; 2013–2015: DST centi-T, Star-Oddi, Iceland; resolution 0.032°C). Loggers were set to record temperature at 5-min intervals. Each temperature logger was calibrated in an insulated water bath against a high accuracy thermometer (Quat 100, Heraeus, Germany) over a temperature range of 28–42°C in increments of 2°C before implantation. We confirmed the reliability of temperature readings by re-calibrating all loggers after their removal. After calibration, the biologgers measured body temperature to an accuracy of better than 0.1°C.

Locomotor activity (see Supplementary Table S3 for a summary) was recorded using motion-sensitive loggers. In the first year (July 2012 to July 2013), activity was recorded by tri-axial piezoelectrical accelerometers (Actical, Mini-Mitter Corporation, United States), which were sensitive to forces of >0.05 *g* and were set to record whole-body movements over full 5-min periods. In the following years (2013–2015), aardvark locomotor activity was recorded over 10-s periods at 5-min intervals using custom-designed activity biologgers (MLOG_AT1, Sigma Delta Technologies, Australia) with a tri-axial accelerometer sensitive to forces of >0.04 *g*. These devices had previously been validated against visual observations of a free-living primate (McFarland et al., 2013).

### Time of Emergence and Return to Burrows

We set up camera traps (MMS wireless scouting camera, LTL-6210MC HD series, Ltl Acorn, China) outside aardvark burrows to determine the exact time of emergence from and return to a burrow after foraging (if the aardvark returned to the same burrow from which it had emerged). Between July 2013 and September 2015, we located each aardvark inside its burrow several times per month using VHF tracking, and placed camera traps at burrow entrances, either behind the burrow entrance or downwind of the burrow to minimise disturbance of the aardvark inside. Because each aardvark changed burrows frequently, we could not use a fixed camera trap system. In total, we obtained recordings of 387 emergences and 109 returns of an aardvark to a burrow (see Supplementary Table S4 for details). We analysed all camera trap footage manually to determine time of emergence and return to burrows.

To determine whether activity loggers could reliably indicate the beginning and end of aboveground activity of aardvarks, we time-matched logger data to camera trap footage of an aardvark’s times of emergence from a burrow, or an aardvark’s time of return to a burrow after foraging. Occasionally after emergence from a burrow, an aardvark remained at the burrow to rest before initiating foraging; in these cases, the time of emergence was identified as the time at which the aardvark started foraging. For each data point from the activity logger, we calculated the median of six values before and six values after this data point (i.e., median activity over a duration of 65 min). If the median exceeded 5% of the maximum activity recorded for each individual, we considered the aardvark to be “active”; if not, it was considered to be “inactive.” We classified the time-stamp of the data point at which the 65-min median activity level changed from “inactive” to “active” to be the time of emergence, whereas the reverse change from “active” to “inactive” marked the time of the aardvark’s return to a burrow. To ensure accuracy of our calculated times of emergence from and return to burrows, we validated calculated times against the actual times obtained from camera trap footage where logger data were available at the same time as camera trap footage (*N* = 176).

### Data Analyses

Some data loggers failed, thereby reducing our sample size to *n* = 10 aardvarks for body temperature and *n* = 9 aardvarks for activity data. We excluded body temperature and activity data recorded on capture days and for 2 days after capture attempts from all analyses. To account for differences in the sensitivity of individual activity loggers, each 5-min reading of activity was expressed as a percentage of the maximum activity reading (% of maximum) for each logger while implanted in a study animal (McFarland et al., 2013). For each aardvark over each 24-h period, we calculated the minimum, maximum, and amplitude (difference between maximum and minimum) of body temperature rhythm. To compare 24-h patterns of aardvark body temperature and activity during the hottest and coldest months of a drought year with a non-drought year, we calculated mean ± SD of hourly body temperature and activity recordings averaged across all aardvarks over 4 months of interest, namely February 2013 (drought summer), August 2013 (winter following drought), February 2014 (non-drought summer), and August 2014 (winter following non-drought summer). We obtained air temperature at the time of emergence and at the time of return of each study aardvark per day (*n* = 1 to 5), and averaged the air temperature coinciding with time of emergence and time of return between these aardvarks for each 24-h period.

We used a series of generalised linear mixed-effect models (GLMMs) to determine the effects of environmental variables on aardvark body temperature patterns. We used each 24-h period as a sampling unit in the GLMMs, and added a first order autocorrelation structure to account for temporal autocorrelation. We included aardvark identity as a random factor. The dependent variable was minimum or maximum or amplitude of the 24-h body temperature rhythm. Independent variables included photoperiod, minimum or maximum or range of 24-h air temperature, and vegetation greenness as a proxy of resource abundance for aardvark prey. This vegetation greenness index consisted of moderate-resolution imaging spectroradiometry (MODIS)-derived time-series enhanced vegetation index (EVI) data for the Gordonia duneveld vegetation (Tokura, 2016). EVI was measured every 16 days and averaged per month. We previously have shown that EVI as an index for aardvark prey’s food availability correlated with aardvark body condition (Weyer, 2018). We tested for multicollinearity of independent variables within each model using variance inflation factors (VIF) and ensured that all VIF <2, based on a conservative cutoff (Zuur et al., 2010).

To assess changes in activity patterns, we calculated the duration of activity between time of emergence from and return to the burrows each day. On occasion, aardvarks did not emerge from their burrows and remained inactive (see Supplementary Table S3); foraging duration was assigned a zero value for these days. For each aardvark, we also calculated the proportion of each active phase that occurred between sunrise and sunset, to quantify diurnal activity of aardvarks. We used a series of GLMMs to determine which independent variables influenced duration of active phase, proportion of diurnal activity, time of emergence and time of return to the burrows relative to time of sunset and sunrise, respectively. Independent variables included minimum 24-h body temperature, minimum 24-h air temperature, EVI, and photoperiod (time between sunrise and sunset) or time of sunset or time of sunrise. Again, we used each 24-h period as a sampling unit in the GLMMs, and added a first order autocorrelation structure to account for temporal autocorrelation. We confirmed VIF <2 for all independent variables in each model and aardvark identity was included as random factor. For time of emergence and return, camera trap data supplemented the dataset on days when no logger data were recorded (May to September 2015).

We used Python 2.7.13 (Python Software Foundation, United States) for the determination of daily time of emergence and time of return of aardvarks from their burrows. We used R statistical computing environment (R Core Team, 2018) with the package lme4 (Bates et al., 2015) to perform the GLMM analysis, and Excel 2016 (Microsoft Corporation, United States), Python 2.7.13 and Prism 5 (GraphPad Software, Inc., United States) to produce graphs. We present data as mean ± SD, and considered *P* < 0.05 to be statistically significant.

## Results

### Environmental Conditions

**FIGURE 1:**
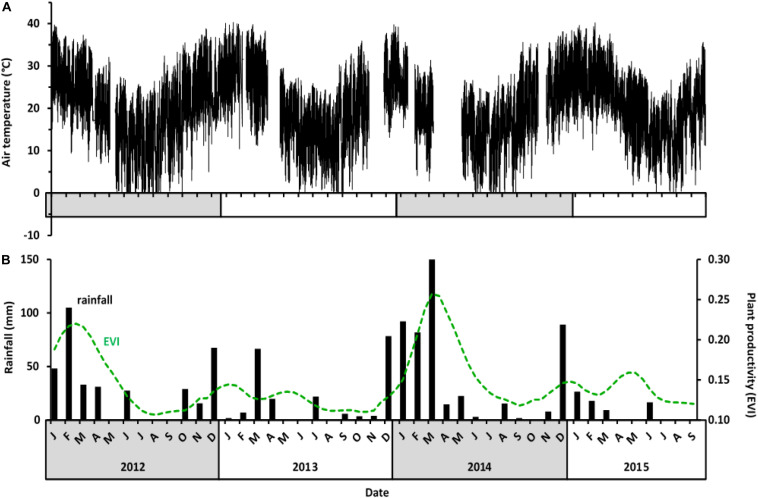
Climate variables at Tswalu from July 2012 to September 2015. **(A)** Air temperature (°C) at 30-min-intervals. Temperature data were not available for periods in February 2013, November 2013, January 2014, and April 2014 because of weather station failure. **(B)** Monthly rainfall (black bars) and plant productivity presented as enhanced vegetation index (EVI; dotted green line) for the Gordonia duneveld vegetation at Tswalu.

Air temperatures at Tswalu varied seasonally, but interannual differences were small (Figure 1A), with annual summer air temperature maxima ranging between 37.9 and 39.9°C, whereas annual winter minima ranged between −1.5 and −4.4°C. In contrast, the amount and timing of rainfall showed high intra- and inter-annual variability, which in turn influenced vegetation greenness (Figure 1B). Total annual rainfall over 33 years, including 30 years before the study commenced, was highly variable (1982–2015: median 228 mm, 1st and 3rd quartile 178 and 270 mm, range 138–392 mm). In the year before our study started (July 2011–June 2012), rainfall was ∼340 mm, i.e., within the higher quartile range, and resulted in high vegetation productivity during that summer (∼85% of the maximum during our study) and the following winter. In year 1 of the study (July 2012–June 2013), rainfall was 196 mm, i.e., between the lower quartile range and the median, and no rain occurred during the hottest months of the summer. Vegetation productivity during the summer 2012–2013 and the following winter 2013 (termed “drought summer” and “drought winter”) was less than ∼54% of the maximum EVI during our study. In year 2 (July 2013 to June 2014), rainfall was 461 mm, within the higher quartile range, and spread over 4 months. The high rainfall of the 2013–2014 summer resulted in the maximum vegetation productivity (EVI = 0.26) which only slowly declined until July 2014 even though rainfall virtually ceased after March. In year 3 (July 2014 to June 2015), rainfall was 172 mm, i.e., within the lower quartile range of the 33-year period, resulting in a slightly lower vegetation productivity that prevailed until year 4 (July to September 2015, when the study ended). Although the summer of year 3 (notably February 2015) was dry and EVI low, the rainfall was not delayed as long as it was during the summer of year 1, so the reduction in EVI was not as drastic, declining to ∼62% of the maximum.

### Patterns of Aardvark Body Temperatures

**FIGURE 2:**
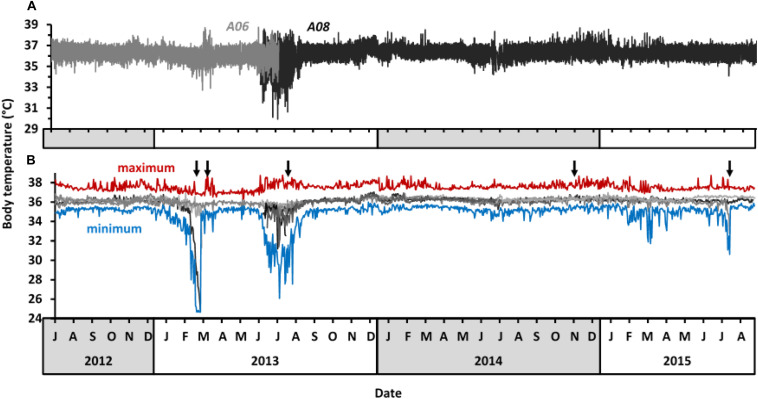
Body temperature of study aardvarks at Tswalu between July 2012 and September 2015. **(A)** 5-min body temperature records of two representative study aardvarks at Tswalu (*A06* and *A08*). **(B)** Overview over the 24-h body temperature profile of all study aardvarks. Grey lines represent mean 24-h body temperature of different individuals, with the individuals from panel **(B)** in the same colours as in panel **(A)**. The upper red and lower blue lines display the absolute maximum and absolute minimum body temperature, respectively, of all study aardvarks within each 24-h period to indicate the total range of body temperatures reached in aardvarks. Black arrows indicate when study aardvarks died.

Body temperatures of study aardvarks ranged from 24.7 to 38.8°C (Supplementary Table S5). The greatest amplitude of 24-h body temperature rhythm was 11.7°C, recorded in aardvark *A11* on 30 July 2013, when its body temperature ranged from 26.1 to 37.8°C within eight hours. The recordings at five-minute intervals of body temperature of two representative aardvarks (*A06* and *A08*) throughout the study period are shown in Figure 2A. Periods of higher than usual variability in the amplitude of 24-h body temperature rhythm were evident in late summer 2012–2013 (notably March) and in winter 2013 (notably July to September). Similar patterns were observed in the mean, minimum, and maximum 24-h body temperatures of all study aardvarks (Figure 2B), with periods of the highest variability coinciding with aardvark deaths (Figure 2B, black arrows). Body temperature patterns of those aardvarks that survived the summer drought and the subsequent 2013 winter recovered to stable values with low variability of mean 24-h body temperature during the spring months, and most aardvarks maintained that state from October 2013 onward until the end of the study in September 2015. An exception was one aardvark that displayed pronounced variability in body temperature during the dry summer in February 2015, and again in winter 2015, before it died in late July 2015.

The GLMM results indicate that minimum 24-h body temperature of aardvarks was associated positively with minimum 24-h air temperature, photoperiod and with EVI (an index for food availability for aardvark prey; Table 1A). Maximum 24-h body temperature of aardvarks was associated positively with photoperiod and negatively with maximum 24-h air temperature, but not associated with EVI (Table 1B). Though significant, the influence of minimum and maximum air temperature on body temperature was trivial (β < 0.01). The amplitude of the 24-h body temperature rhythm was associated negatively with photoperiod and with EVI, but not associated with the range of 24-h air temperature (Table 1C), indicating that days with large fluctuations in air temperature were not associated with large fluctuations in body temperatures.

**TABLE 1:** GLMM results showing effects of photoperiod (hours between sunrise and sunset), minimum or maximum or range of air temperature (°C), and EVI of Gordonia duneveld vegetation (an index of vegetation greenness and thus food availability for aardvark prey) at Tswalu on **(A)** minimum, **(B)** maximum, and **(C)** amplitude of the body temperature rhythm (°C) for each 24-h period between July 2012 and September 2015.

Body temperature (*N* = 3749)	Variable	β ± SE	*t*	*P*
(A) 24-h minimum	Minimum air temperature	0.007 ± 0.002	3.4	<*0.001*
	EVI	3.9 ± 2.0	2.0	<*0.05*
	Photoperiod	5.4 ± 1.4	3.7	<*0.001*
	intercept	31.5 ± 0.8	39.5	<*0.001*
**(B)** 24-h maximum	Maximum air temperature	−0.004 ± 0.002	–2.2	<*0.001*
	EVI	0.9 ± 1.2	0.7	0.47
	Photoperiod	1.7 ± 0.9	2.0	*0.04*
	Intercept	36.4 ± 0.5	80.7	0.48
**(C)** 24-h amplitude	Range of air temperature	0.004 ± 0.003	1.7	0.10
	EVI	−6.9 ± 1.2	–6.0	<*0.001*
	Photoperiod	−3.8 ± 0.8	–5.0	<*0.001*
	Intercept	5.1 ± 0.4	10.7	<*0.001*

*Aardvark identity was included as a random factor (*n* = 10 aardvarks). Significant *P*-values are italicised for ease of reference.*

### Activity Patterns

**FIGURE 3:**
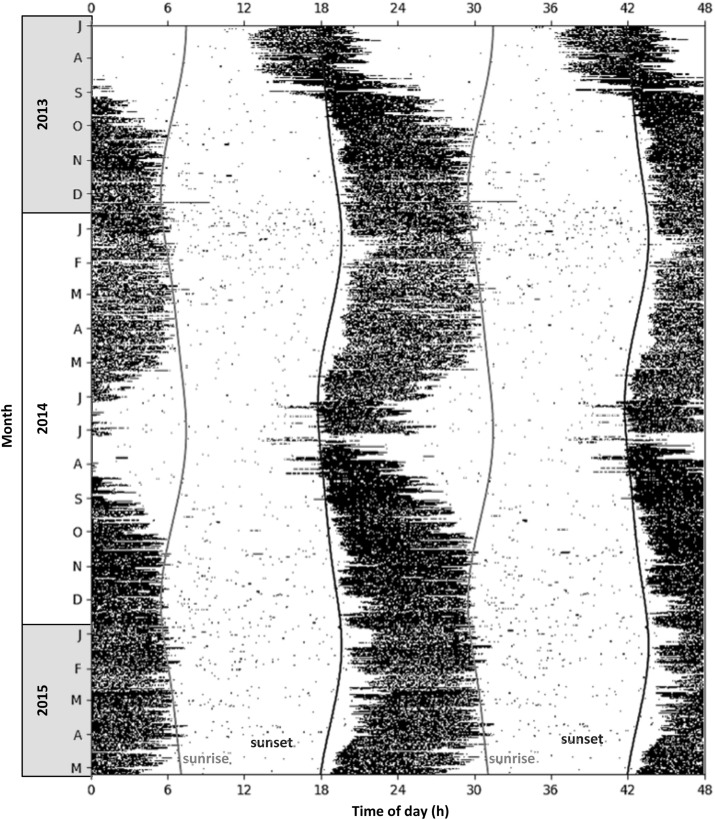
Actogram showing locomotor activity of a single representative aardvark (*A08*) at Tswalu, free-ranging in its natural environment, for the period July 2013 to May 2015 (*N* = 675 consecutive days). Days are stacked vertically, data are displayed in 5-min intervals. Black bars represent periods when the aardvark was active (≥5% of maximum activity); white areas represent periods when the aardvark was inactive (<5% of maximum activity); dark grey line is the time of sunset; light grey line is the time of sunrise. For better visualisation of seasonal shifts in nychthemeral rhythms, activity is plotted over 48 h, with two consecutive 24-h periods displayed beside each other.

**FIGURE 4:**
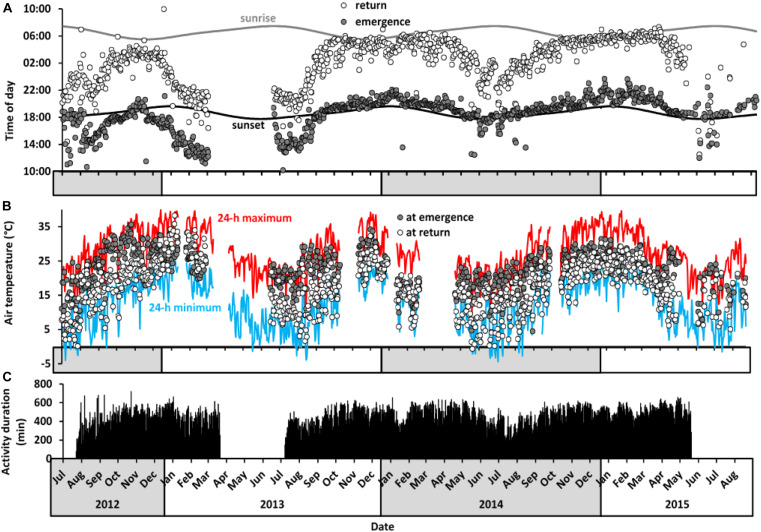
Long-term activity patterns of aardvarks at Tswalu between July 2012 and September 2015. **(A)** Daily time of emergence and return to burrow of all study aardvarks in relation to times of sunrise and sunset. Values are averages for all aardvarks (*n* = 1 to 5 aardvarks) per day (*N* = 1,032 days). **(B)** Air temperature at time of emergence from (grey circles) and return to (white circles) the burrows, averaged across all aardvarks per day. The blue line represents minimum 24-h air temperature and the red line represents maximum 24-h air temperature. Temperature data were not available for periods in February 2013, November 2013, January 2014, and April 2014 because of weather station failure. **(C)** Duration of 24-h activity averaged across study aardvarks. Activity logger data were not available between March and July 2013 and after June 2015 because of failure of data loggers and deaths of several study aardvarks (see Supplementary Table S1 for details).

Locomotor activity of aardvarks over 24-h periods was biphasic, with activity typically high in the scotophase and low in the photophase, but with brief inactive bouts during the active phase, and brief active bouts during the inactive phases (as shown for a representative aardvark *A08*, Figure 3). Patterns of activity of aardvarks varied intra- and inter-annually (Figures 3, 4). Aardvarks were generally active for ∼8 h, but shortened their active phases to ∼6 h during winters (Figure 4C). Late in our study period, aardvarks were exclusively nocturnal, typically emerging at sunset and returning to their burrows at sunrise (Figure 4A). However, early in our study period, there was substantial diurnal activity, accompanied by early returns to the burrows (Figure 4A). Occasionally aardvarks emerged as early as 8 hours before sunset and occasionally they returned to their burrows before sunset, so they had entirely diurnal activity periods. In spite of the variation in times of emergence and return, the duration of the activity period was remarkably constant, except during the winter months, when early returns to the burrows led to curtailed activity periods (Figure 4C). Shifts of the times of the start and end of activity were not synchronised, resulting in a shortening of the active phase coinciding with a shift to diurnal activity (Figure 4).

There was no particular air temperature at which aardvarks emerged from their burrows or returned to them (Figure 4B). The range of air temperatures at which aardvarks emerged (–3.2 to 36.9°C) or returned (–2.4 to 39.4°C) to burrows varied seasonally, and was only slightly narrower than the full range of air temperatures experienced at Tswalu (Figure 4B). Air temperatures usually were higher at the time of emergence than at the time of return, except from January to March 2013, when aardvarks had increased diurnal activity.

The GLMM analyses of variables affecting aardvark activity patterns indicate that the daily duration of the active phase of aardvarks was associated strongly and positively with photoperiod and EVI, and weakly but positively with minimum 24-h body temperature and minimum 24-h air temperature (Table 2A). The proportion of activity that took place diurnally was associated inversely with minimum 24-h body temperature, minimum 24-h air temperature, photoperiod and EVI (Table 2B). Time of aardvark emergence from a burrow relative to sunset was associated positively and strongly with minimum 24-h body temperature and EVI, but not associated with time of sunset or minimum 24-h air temperature (Table 2C). Time of return to a burrow relative to sunrise was associated strongly and positively with EVI and minimum 24-h body temperature, strongly and negatively with time of sunrise, and positively but weakly with minimum 24-h air temperature (Table 2D).

**TABLE 2:** GLMM results showing effects of minimum 24-h body temperature (°C), minimum 24-h air temperature (°C), photoperiod (hours between sunrise and sunset) or time of sunset or time of sunrise, and EVI of Gordonia duneveld vegetation (an index of vegetation greenness and thus food availability for aardvark prey) at Tswalu, on **(A)** duration of the active phase (hours), **(B)** proportion of the daily active phase spent active during daylight hours, **(C)** time of emergence from burrows relative to sunset, and **(D)** time of return to burrows relative to sunrise for each 24-h period between July 2012 and September 2015.

Activity	Variable	β ± SE	*t*-value	*P*
(A) Duration of the	Minimum body temperature	0.008 ± 0.002	3.3	< *0.001*
active phase (*N* = 2,254)	Minimum air temperature	0.002± < 0.001	5.4	< *0.001*
	Photoperiod	0.19 ± 0.07	2.8	*0*.*005*
	EVI	0.27 ± 0.07	3.7	< *0.001*
	Intercept	−0.11 ± 0.07	–1.4	0.15
**(B)** Proportion of	Minimum body temperature	−5.2 ± 0.6	–9.0	< *0.001*
the active phase diurnal	Minimum air temperature	−0.18 ± 0.08	–2.2	*0*.*03*
(*N* = 2,254)	Photoperiod	−41.8 ± 19.9	–2.0	*0*.*04*
	EVI	−63.7 ± 25.2	–2.5	*0*.*01*
	Intercept	239.0 ± 22.4	10.7	< *0.001*
**(C)** Time of emergence	Minimum body temperature	0.39 ± 0.07	5.7	< *0.001*
(*N* = 2,243)	Minimum air temperature	0.014 ± 0.009	1.5	0.12
	Time of sunset	4.3 ± 5.1	0.8	0.41
	EVI	7.6 ± 3.4	2.2	0.03
	Intercept	−19.4 ± 4.4	–4.4	< *0.001*
**(D)** Time of return	Minimum body temperature	0.31 ± 0.08	3.8	< *0.001*
(*N* = 2,243)	Minimum air temperature	0.05 ± 0.01	4.8	< *0.001*
	Time of sunrise	−77.0 ± 5.5	–14.2	< *0.001*
	EVI	22.3 ± 3.8	6.0	< *0.001*
	Intercept	0.97 ± 3.51	0.3	0.78

*No activity data were available from March to June 2013. For time of emergence and return, camera trap data were included in the dataset on days on which no biologger data were recorded (May to September 2015). Aardvark identity was included as a random factor (*n* = 7 aardvarks). Significant *P*-values are italicised for ease of reference.*

### Patterns of 24-h Body Temperature and Activity

**FIGURE 5:**
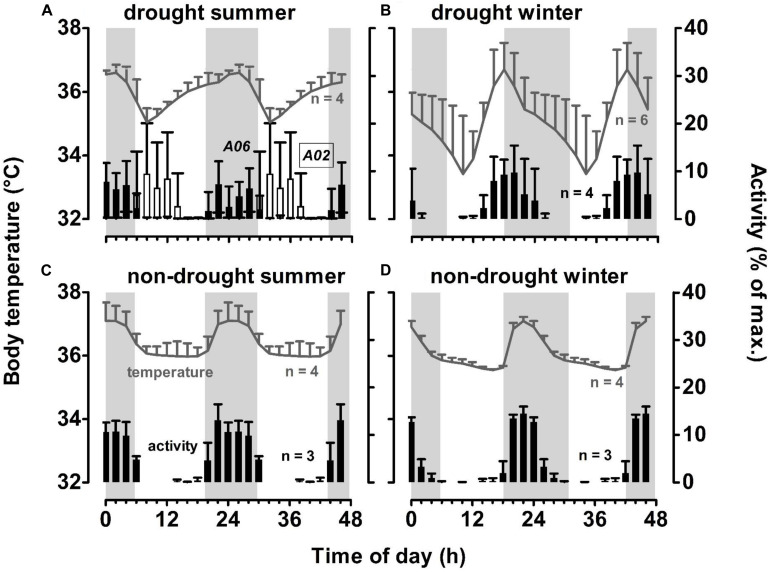
Patterns of 24-h activity and body temperature of study aardvarks, double-plotted for better visualisation, during a month of a drought summer (February 2013, panel **(A)**, the following drought winter (August 2013, panel **(B)**, during a non-drought summer (February 2014, panel **(C)**, and the following non-drought winter (August 2014, panel **(D)** at Tswalu. Grey areas indicate night time. Error bars indicate SD between individuals. Note that on panel **(A)**, activity patterns of two aardvarks for which data were available were plotted individually to display the difference between an aardvark that survived the drought summer (*A06*, filled bars), and one that died in the following month (*A02*, empty bars). Mean minimum 24-h air temperatures during the winter months were similar (4.5°C in August 2013; 7.6°C in August 2014). Mean maximum 24-h air temperatures differed between the summer months, with the drought summer up to 10°C hotter than the non-drought summer month (36.5°C in February 2013; 26.0°C in February 2014.

To visualise how low resource availability during the drought influenced body temperature and activity of our aardvarks, we graphed (Figure 5) aardvark activity and body temperature, as a function of time of day, for a winter and summer month, during a drought year (rainfall in the lowest quartile of a 33-year record) and during a non-drought year (rainfall in the highest quartile of a 33-year record). In the absence of drought, body temperature assumed a bimodal rhythm, with elevated temperatures at night, coinciding with aardvarks’ nocturnal activity, and a lower plateau (February 2014, summer, Figure 5C) or depression with slight decline (August 2014, winter, Figure 5D) coinciding with daily inactive phases inside the burrows. Over the winter month, which followed a non-drought summer, aardvarks shifted their activity to slightly earlier times of day than in summer, becoming partly diurnal, and the nocturnal peak in body temperature occurred somewhat earlier (Figure 5D).

Those body temperature profiles were substantially distorted during a drought. The profiles of body temperature no longer were bimodal but more cosinor. The 24-h maxima of body temperature differed little from those in the absence of drought, but in the winter month (August 2013, Figure 5B), the 24-h maximum occurred before sunset, coinciding with a shift in activity pattern as aardvarks emerged earlier, with activity starting to increase during the late morning, and peaking during the early evening. During the drought summer month (February 2013, Figure 5A), the two aardvarks for which we retrieved activity data had different activity patterns; one became diurnal and the other remained largely nocturnal. The average body temperature of these two, plus two other aardvarks for which we retrieved body temperature data but not activity data during this particular month, continued to peak at night, but now after a gradual increase beginning soon after sunrise. The stable body temperatures that occurred during the day in the absence of a drought disappeared, presumably because some aardvarks were not in the burrow, and body temperature dipped to a nadir occurring a few hours after sunrise. In the drought winter month (Figure 5B), that nadir, averaged for six aardvarks for which we retrieved body temperature data, was below 34°C. Variability in body temperature between aardvarks, as indicated by the standard deviation, was much higher during the drought winter month than in the non-drought winter month, when aardvarks had remarkably similar body temperatures. In the summer months, drought had little influence on that variability.

The mean amplitude of the 24-h rhythm of aardvark body temperature during a non-drought summer month (1.8 ± 0.3°C) increased during a drought summer to 2.3 ± 0.4°C, which was similar to that evident in a non-drought winter month (2.1 ± 0.1°C). Mean amplitude of 24-h body temperature rhythm in a drought winter month (4.7 ± 1.3°C) was more than double the amplitude during the equivalent month in the absence of a drought.

## Discussion

**FIGURE 6:**
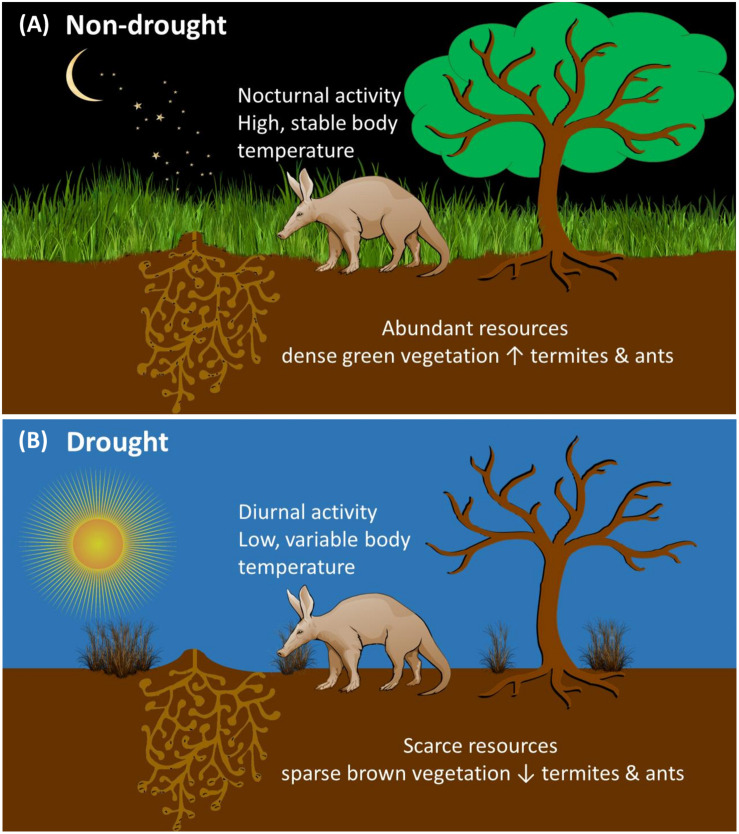
Pictogram illustrating the interplay of different factors influencing aardvark body temperature and activity **(A)** during a non-drought period with green grass and plenty of prey termites and ants available, aardvarks are active at night and maintain high and stable body temperature rhythms; **(B)** during a drought period with sparse brown grass and few prey insects deep in burrows aardvarks are active during the daytime and display low and variable body temperature rhythms.

Our long-term study over ∼3.5 years assessed intra-and inter-annual variability in aardvark body temperature and activity responses to fluctuations in environmental conditions in the semi-arid Kalahari and allowed us to elucidate differences between drought and non-drought years. During a drought year, vegetation was scarce. Aardvarks were partly diurnal during the drought summer as well as during much of the subsequent winter, and displayed minimum 24-h body temperatures as low as 24°C, resulting in exaggerated heterothermy. Several aardvarks died during the drought summer and the following winter after exhibiting large amplitudes of the 24-h body temperature rhythm and diurnal foraging activity. In comparison, during non-drought years, when above-average rainfall generated high vegetation productivity and thus likely high availability of aardvark prey (Weyer, 2018), aardvarks remained nocturnal throughout the summer and most of the winter. Rather than extending their foraging duration in an attempt to obtain more food, aardvarks shortened their foraging duration during the drought and winter, most notably during a winter following drought. Our data showed that, of the variables that we measured, the key driver of changes in body temperature and activity in our Kalahari aardvarks was EVI. In our GLMMs, EVI (a measure of vegetation greenness) was associated positively with minimum 24-h body temperature, and very strongly and inversely with amplitude of the 24-h body temperature rhythm, an index that we have proposed for the well-being of mammals (Hetem et al., 2016). EVI also was associated positively with the duration of the aardvarks’ foraging activity and with the time of emergence and return to the burrows, which resulted in an increase in the proportion of diurnal activity that took place when vegetation was brown. Moreover, minimum 24-h body temperature was a relevant driver of aardvark activity, such that the lower their minimum 24-h body temperature was, the more likely aardvarks were to become diurnal, and to emerge from and return to their burrows earlier. We conclude that the aberrations that we observed in body temperature and activity patterns during droughts were mainly related to energy deficits, and the aardvarks behaved in a way that reduced the thermoregulatory cost of those energy deficits (see Figure 6). Similar declines in minimum 24-h body temperature have been observed in other large mammals that experienced an energy deficit (Hetem et al., 2016). For example, Arabian oryx (*Oryx leucoryx*) showed low minimum 24-h body temperatures throughout the dry, resource-scarce period, irrespective of ambient temperatures (Hetem et al., 2010).

It was not low EVI itself that resulted in energy deficits during droughts. Vegetation greenness provided a measure of resource availability because the aardvarks’ primary prey item, the harvester termite *Hodotermes mossambicus* (Weyer, 2018), consumes grass (Nel and Hewitt, 1969; Symes and Woodborne, 2011). The higher the EVI, the more abundant or nutritious would have been the termites, and the greater the amount of dietary energy, and dietary water, available readily to the aardvarks. The aardvarks consequently lost body condition when EVI was low (Weyer, 2018). Loss of body fat would have compromised the capacity of the aardvarks to respond metabolically to cold. If they were to respond, aardvarks would have to do so behaviourally by retreating to warmer burrows and exploiting ambient warmth during the day. That they did by engaging in diurnal activity, and the lower their minimum 24-h body temperatures were, the greater was the proportion of their active phase that took place diurnally. Many aardvarks died at our study site at the end of the 2013 summer drought (Rey et al., 2017), with some aardvarks shifting activity entirely to the daylight hours in the weeks leading up to their deaths. Those deaths and failure of some of our biologging devices reduced our sample size and we did not have continuous measurements of body temperature and activity in the same individuals over the 3.5-year study, but we are confident that our GLMM analyses were robust. Indeed, these analyses have allowed us to present the longest and most comprehensive study on the ecophysiology of free-living aardvarks to date.

Aardvarks were affected adversely by reduced prey availability not only during the 2013 summer drought itself, but afterwards, because surviving aardvarks took a long time to improve their body condition (indicated by return of homeothermic body temperature patterns) after the drought winter. The single individual (*A06*, Figure 2) for which data are available for the autumn after the 2013 drought summer did not show diurnal activity and had only short bouts of heterothermy during the summer drought compared to other aardvarks, and showed a rapid return to stable thermoregulation following the onset of summer rains, but a second decline in minimum body temperature and longer-lasting heterothermy during the subsequent cold and dry winter. During this winter (2013), additional aardvarks died (including one of our study aardvarks) and no study aardvark maintained stable body temperature at night (mean amplitude of the 24-h body temperature rhythm during August 2013 was 4.7 ± 1.3°C; absolute range 24.7 to 38.8°C). The 11.7°C amplitude of the 24-h body temperature rhythm in a surviving individual during that winter is the largest recorded in any large, free-living mammal to date, larger than that previously reported for aardvarks during the summer drought (8.6°C; Rey et al., 2017) or for Arabian oryx in the harsh Arabian Desert (7.7°C; Hetem et al., 2010). The variable minimum 24-h body temperatures of our aardvarks are incongruent with the controlled downregulation of body temperature as a result of a reduction of the hypothalamic set-point, as in torpor (Heldmaier and Ruf, 1992). Rather than a regulated response, the loss of body condition and high mortality rate associated with lowered 24-h minimum body temperatures of our aardvarks imply that the animals were experiencing an energy deficit. Like our aardvarks during non-drought conditions, free-living aardvarks during winter and summer in the Karoo, South Africa displayed a bimodal 24-h rhythm of body temperature with lower body temperature during the day when the aardvarks were inactive inside their burrows, and higher body temperatures at night when they were foraging actively (Taylor and Skinner, 2004). Taylor and Skinner (2004) suggested that the endogenous heat produced by intense digging might restrict aardvark foraging to the cooler night-time during hot periods whereas sensitivity to cold might force aardvarks to shift their foraging activity earlier; they claimed that aardvarks avoided air temperatures below 2°C. Our data do not support those suggestions. On occasion, our aardvarks still were active at air temperatures below freezing (compare panels **A** and **B** of Figure 4), and we observed them engaged in digging activity during the heat of the day in summer.

When aardvarks had low minimum 24-h body temperatures, they increased the time they spent active during daylight hours but decreased their total foraging duration. Optimal foraging theory proposes that an animal should maximise energy intake while taking into account all potential costs and benefits of foraging (Krebs, 1980; Pyke, 1984), such that the animal would continue to forage as long as energetic benefits exceed the likely costs of foraging, including predation risk and energy costs (Lima and Dill, 1990). When resources become limited, an animal might either increase the duration or intensity of its foraging activities to locate sparse resources (Therrien et al., 2008; Clutton-Brock et al., 2009; Podolski et al., 2013), or decrease activity to conserve energy (O’Donnell, 2000; Christian and Geiser, 2007). During a drought summer and resource-limited winters, when prey availability was reduced, our aardvarks did not compensate by foraging for longer, likely to avoid energetic costs incurred by foraging, and consequently lost body condition, sometimes lethally.

The activity pattern of our aardvarks in the Kalahari during non-drought years, when it was not dominated by the need to survive starvation, resembled that of aardvarks in the Karoo (Van Aarde et al., 1992; Taylor, 1998), in that when days were long in summer (and therefore the nights were short), the aardvarks counterintuitively were active for longer (∼8 h per day), and almost exclusively nocturnal. During short days in winter, both our aardvark and those in the Karoo (Taylor, 1998) reduced the duration of activity (∼6 h per day) and aardvarks emerged from and returned to their burrows earlier in the day, particularly when resource availability (as indexed by EVI) was low.

In terms of aardvarks timing their activity according to their prey availability, optimal foraging theory predicts that a species would coordinate the timing of its foraging activities with the times of the day at which their prey is most accessible, to maximise energy intake. Harvester termites, a key prey item of Kalahari aardvarks (Weyer, 2018), occur in deep (up to 7 m) underground hives (Hartwig, 1965). To harvest grass (Nel and Hewitt, 1969; Symes and Woodborne, 2011), these termites must undergo daily vertical migrations from the deep hive area and move to the upper, warmer soil levels during the day, to warm up in anticipation of their daily foraging. Thus, in winter, termites might aggregate near the surface several hours before emerging to forage during the daytime, which might explain a shift in aardvark foraging to earlier times in winter. In summer, the termites would be expected to migrate toward the upper levels of the soil in the evening, in anticipation of their night-time foraging, which would match the nocturnal activity of the aardvarks in summer. However, aardvarks at Tswalu became mostly diurnal during drought winters but less so during non-drought winters, thus an adaptation of their active phase to the timing of termite activity was unlikely to have been the explanation for the seasonal shift in aardvark activity. Similarly, in the Karoo, aardvark activity was independent of prey activity as the aardvarks were nocturnal but their predominant prey ants *Anoplolepis custodiens* (Taylor et al., 2002) were predominantly diurnal (Lindsey and Skinner, 2001). Moreover, harvester termites also have been reported to forage diurnally even at high temperatures (Nel, 1970), substantiating the argument that aardvark activity is independent of that of prey activity. That our aardvarks could forage at night or in the day implies that termites were available to them at all times.

The main factor that influenced the proportion of activity that was diurnal in our aardvarks was their minimum 24-h body temperature, proposed as an index of nutritional state (Rey et al., 2017). Our aardvarks switched to more-diurnal activity after they no longer had sufficient energy to maintain body temperature at night. During periods of negative energy balance, other nocturnal mammals also shift their activity to the daytime, both in the laboratory (Hut et al., 2011; Van der Vinne et al., 2014, 2015) and in the wild (Lockard, 1978; Boal and Giovanni, 2007). The shift to diurnality in response to an energy deficit is more pronounced under cold conditions (Van der Vinne et al., 2014), when metabolic costs associated with thermoregulation are highest. Such plasticity of the mammalian circadian system is thought to be an adaptive mechanism that allows nocturnal mammals to conserve their energy (up to 10% in some species, Van der Vinne et al., 2015), thereby enhancing their survival (Van der Vinne et al., 2019). However, if the shift to diurnality occurs when ambient temperatures are high (Van der Vinne et al., 2014), as it did in our aardvark during the summer drought, it would increase their exposure to heat.

The suite of adaptive responses available to aardvarks that has been sufficient to ensure their survival in the past may not be sufficient in the future. In semi-arid zones with summer rainfall, such as the Kalahari, availability of resources is dictated primarily by the timing and amount of rainfall (Noy-Meir, 1973; Hawkins et al., 2003), and delayed onset of annual rains, as projected in the Kalahari under climate change (Shongwe et al., 2009), results in low vegetation productivity at a time when conditions are getting hotter and drier. Summer droughts combined with heat waves, as observed in the summer of 2013, will become increasingly frequent in southern Africa as a result of climate change (Niang et al., 2014; Phaduli, 2018), as has already been evident during the past decade (Jordaan, 2012; Agri-SA, 2019; Ngqakamba, 2019). Such climatic changes and consequent changes in resource availability might severely impact the persistence of aardvarks if their physiological and behavioural capacity to adapt is exceeded. Extirpation of aardvarks, key ecosystem engineers that provide thermal refugia to dozens of vertebrates (Whittington-Jones et al., 2011), might have severe cascading effects for biodiversity in the Kalahari ecosystem.

## Data Availability Statement

The data sets analysed for this study are available on request to the corresponding author.

## Ethics Statement

All procedures were approved by the Department of Environment and Nature Conservation of South Africa (Northern Cape Province Government, permits no. FAUNA 1000/2013, 1000/2/2013, and 1001/2013) and the Animal Ethics Screening Committee of the University of the Witwatersrand (clearance certificate no. AESC 2011/10/04 and AESC 2013/29/05).

## Author Contributions

All authors contributed to study design, field work and data interpretation, edited the manuscript, approved the final version of the manuscript, and agreed to be held accountable for the content therein. AH and LM carried out surgical procedures. NW and RH analysed the data. NW drafted the manuscript.

## Conflict of Interest

The authors declare that the research was conducted in the absence of any commercial or financial relationships that could be construed as a potential conflict of interest.

## Footnotes

1 https://giovanni.gsfc.nasa.gov/giovanni/

2 http://staging.timeanddate.com
