# Supplementary material for: Increased Diurnal Activity Is Indicative of Energy Deficit in a Nocturnal Mammal, the Aardvark
Source: Front Physiol. 2020 Jul 7;11:637. doi: 10.3389/fphys.2020.00637 (PMC7358442; doi:10.3389/fphys.2020.00637)
Supplement: Supplementary file 1 [file Table_1.docx]

**Supplementary Material**

**Supplementary Table 1.** Gantt chart indicating time periods for which activity and body temperature data were obtained from each study aardvark at Tswalu between July 2012 and September 2015. ID − aardvark identity; light grey - activity data; dark grey - body temperature data; blank fields - no data available; * − logger implantation; Ɨ − death of the aardvark; Ω − logger not retrieved; # − logger failure; x − logger removal.

|  | **2012** | | | | | | **2013** | | | | | | | | | | | | **2014** | | | | | | | | | | | | **2015** | | | | | | | | |
| --- | --- | --- | --- | --- | --- | --- | --- | --- | --- | --- | --- | --- | --- | --- | --- | --- | --- | --- | --- | --- | --- | --- | --- | --- | --- | --- | --- | --- | --- | --- | --- | --- | --- | --- | --- | --- | --- | --- | --- |
| **ID** | **J** | **A** | **S** | **O** | **N** | **D** | **J** | **F** | **M** | **A** | **M** | **J** | **J** | **A** | **S** | **O** | **N** | **D** | **J** | **F** | **M** | **A** | **M** | **J** | **J** | **A** | **S** | **O** | **N** | **D** | **J** | **F** | **M** | **A** | **M** | **J** | **J** | **A** | **S** |
| *A01* | ***#** |  |  |  |  |  |  | **Ɨ** |  |  |  |  |  |  |  |  |  |  |  |  |  |  |  |  |  |  |  |  |  |  |  |  |  |  |  |  |  |  |  |
|  | ***#** |  |  |  |  |  |  | **Ɨ** |  |  |  |  |  |  |  |  |  |  |  |  |  |  |  |  |  |  |  |  |  |  |  |  |  |  |  |  |  |  |  |
| *A02* | ***** |  |  |  |  |  |  |  |  | **Ɨ** |  |  |  |  |  |  |  |  |  |  |  |  |  |  |  |  |  |  |  |  |  |  |  |  |  |  |  |  |  |
|  | ***** |  |  |  |  |  |  |  |  | **Ɨ** |  |  |  |  |  |  |  |  |  |  |  |  |  |  |  |  |  |  |  |  |  |  |  |  |  |  |  |  |  |
| *A03* | ***** |  |  |  |  |  |  |  |  | **ƗΩ** |  |  |  |  |  |  |  |  |  |  |  |  |  |  |  |  |  |  |  |  |  |  |  |  |  |  |  |  |  |
|  | ***** |  |  |  |  |  |  |  |  | **ƗΩ** |  |  |  |  |  |  |  |  |  |  |  |  |  |  |  |  |  |  |  |  |  |  |  |  |  |  |  |  |  |
| *A04* | ***** |  |  |  |  |  |  |  |  | **Ɨ** |  |  |  |  |  |  |  |  |  |  |  |  |  |  |  |  |  |  |  |  |  |  |  |  |  |  |  |  |  |
|  | ***** |  |  |  |  |  |  |  |  | **Ɨ** |  |  |  |  |  |  |  |  |  |  |  |  |  |  |  |  |  |  |  |  |  |  |  |  |  |  |  |  |  |
| *A05* | ***** |  |  |  |  |  |  |  | **ƗΩ** |  |  |  |  |  |  |  |  |  |  |  |  |  |  |  |  |  |  |  |  |  |  |  |  |  |  |  |  |  |  |
|  | ***** |  |  |  |  |  |  |  | **Ɨ** |  |  |  |  |  |  |  |  |  |  |  |  |  |  |  |  |  |  |  |  |  |  |  |  |  |  |  |  |  |  |
| *A06* | ***** |  |  |  |  |  |  |  |  | **#** |  |  |  |  | **x** |  |  |  |  |  |  |  |  |  |  |  |  |  |  |  |  |  |  |  |  |  |  |  |  |
|  | ***** |  |  |  |  |  |  |  |  |  |  |  |  | **#** | **x** |  |  |  |  |  |  |  |  |  |  |  |  |  |  |  |  |  |  |  |  |  |  |  |  |
| *A07* |  |  |  |  |  |  |  |  |  |  |  |  | ***** |  |  |  |  |  |  |  |  |  |  |  | **x** |  |  |  |  |  |  |  |  |  |  |  |  |  |  |
|  |  |  |  |  |  |  |  |  |  |  |  |  | ***** |  |  |  |  |  |  |  |  |  |  |  | **x** |  |  |  |  |  |  |  |  |  |  |  |  |  |  |
| *A08* |  |  |  |  |  |  |  |  |  |  |  |  | ***** |  |  |  |  |  |  |  |  |  |  |  | **x*** |  |  |  |  |  |  |  |  |  |  | **#** |  |  | **x** |
|  |  |  |  |  |  |  |  |  |  |  |  |  | ***** |  |  |  |  |  |  |  |  |  |  |  | **x*** |  |  |  |  |  |  |  |  |  |  |  |  |  | **x** |
| *A09* |  |  |  |  |  |  |  |  |  |  |  |  | ***#** |  |  |  |  |  |  |  |  |  |  |  |  |  |  |  |  |  |  | **x*#** |  |  |  |  |  | **Ɨ** |  |
|  |  |  |  |  |  |  |  |  |  |  |  |  | ***** |  |  |  |  |  |  |  |  |  |  |  |  |  |  |  |  |  |  | **x*** |  |  |  |  |  | **Ɨ** |  |
| *A10* |  |  |  |  |  |  |  |  |  |  |  |  | ***** |  | **Ɨ** |  |  |  |  |  |  |  |  |  |  |  |  |  |  |  |  |  |  |  |  |  |  |  |  |
|  |  |  |  |  |  |  |  |  |  |  |  |  | ***** |  | **Ɨ** |  |  |  |  |  |  |  |  |  |  |  |  |  |  |  |  |  |  |  |  |  |  |  |  |
| *A11* |  |  |  |  |  |  |  |  |  |  |  |  | ***** |  |  |  |  |  |  |  |  |  |  |  |  |  |  |  | **Ɨ** |  |  |  |  |  |  |  |  |  |  |
|  |  |  |  |  |  |  |  |  |  |  |  |  | ***** |  |  |  |  |  |  |  |  |  |  |  |  |  |  |  | **Ɨ** |  |  |  |  |  |  |  |  |  |  |
| *A12* |  |  |  |  |  |  |  |  |  |  |  |  |  |  |  |  |  |  |  |  |  |  |  |  | ***** |  |  |  |  |  |  |  |  |  |  | **#** |  |  | **x** |
|  |  |  |  |  |  |  |  |  |  |  |  |  |  |  |  |  |  |  |  |  |  |  |  |  | ***** |  |  |  |  |  |  |  |  |  |  |  |  |  | **x** |

**Supplementary Table 2.** Dimensions of implanted devices.

| **Devices** | **Dimensions** | **Mass** |
| --- | --- | --- |
| VHF tracking transmitter | 30 mm Ø, 110 mm length | ~100 g |
| Temperature loggers |  |  |
| iButton | ~55 mm × 45 mm × 10 mm | ∼40 g |
| Star-Oddi | ~15 mm Ø, 46 mm length | ~20 g |
| Activity loggers |  |  |
| Actical | ~35 mm × 35 mm × 15 mm | ~40 g |
| MLOG-AT1 | ~20 mm × 40 mm × 40 mm | ~25 g |

**Supplementary Table 3.** Overview of locomotor activity data recorded with biologgers in each study aardvark at Tswalu between July 2012 and May 2015. ID − aardvark identity; N days recorded − number of days during which activity was recorded; N days active − number of days each aardvark was active for ≥65 minutes; N days inactive − number of days the aardvark was inactive.

| **ID** | **N days recorded** | **N days active** | **N days inactive** |
| --- | --- | --- | --- |
| *A01* | 181 | 181 | 0 |
| *A02* | 227 | 225 | 2 |
| *A03* | − | − | − |
| *A04* | 68 | 65 | 5 |
| *A05* | − | − | − |
| *A06* | 227 | 225 | 2 |
| *A07* | 384 | 382 | 2 |
| *A08* | 672 | 672 | 0 |
| *A09* | − | − | − |
| *A10* | 34 | 32 | 2 |
| *A11* | 478 | 470 | 8 |
| *A12* | 303 | 301 | 2 |

**Supplementary Table 4.** Summary of the total numbers of emergences (N emergences recorded) and returns (N returns recorded) recorded on camera trap for each study aardvark at Tswalu over the period July 2012 to September 2015. ID − aardvark identity.

| **ID** | **N emergences recorded** | **N returns recorded** |
| --- | --- | --- |
| *A01* | − | − |
| *A02* | − | − |
| *A03* | − | − |
| *A04* | − | − |
| *A05* | − | − |
| *A06* | − | − |
| *A07* | 65 | 6 |
| *A08* | 142 | 30 |
| *A09* | 86 | 35 |
| *A10* | 10 | 4 |
| *A11* | 62 | 28 |
| *A12* | 22 | 6 |
| Total | 387 | 109 |

**Supplementary Table 5.** Summary of 24-h body temperature data of all study aardvarks at Tswalu over the period July 2012 to September 2015. ID − aardvark identity; Period − time during which the aardvark was instrumented with a body temperature data logger; N − number of 24-h periods over which body temperature was recorded (excluding capture periods).

| ID | Period | 24-h body temperature (°C) | | | | N | | Died? | | | Circumstances of death | |  |
| --- | --- | --- | --- | --- | --- | --- | --- | --- | --- | --- | --- | --- | --- |
|  |  | **minimum** | **maximum** | **maximum amplitude** |  | |  | | |  |  |  |  |
| *A01* | Jul 2012 to Jan 2013 | − | − | − | − | | | | yes | | | drought | |
| *A02* | Aug 2012 to Mar 2013 | 24.7 | 38.5 | 8.1 | 223 | | | | yes | | | drought | |
| *A03* | Aug 2012 to Mar 2013 | − | − | − | − | | | | yes | | | drought | |
| *A04* | Aug 2012 to Mar 2013 | 31.8 | 38.4 | 4.7 | 237 | | | | yes | | | drought | |
| *A05* | Aug 2012 to Feb 2013 | 34.1 | 38.4 | 3.1 | 197 | | | | yes | | | drought | |
| *A06* | Aug 2012 to Jul 2013 | 32.8 | 38.7 | 4.3 | 365 | | | | no | | | − | |
| *A07* | Jul 2013 to Jul 2014 | 30.7 | 38.4 | 7.1 | 384 | | | | no | | | − | |
| *A08* | Jul 2013 to Sep 2015 | 29.9 | 38.7 | 8.4 | 793 | | | | no | | | − | |
| *A09* | Jul 2013 to Jun 2015 | 30.6 | 38.8 | 7.3 | 752 | | | | yes | | | winter after drought | |
| *A10* | Jul 2013 to Aug 2013 | 27.6 | 38.3 | 9.3 | 34 | | | | yes | | | winter after drought | |
| *A11* | Jul 2013 to Oct 2014 | 26.1 | 38.7 | 11.7 | 476 | | | | yes | | | snake bite? | |
| *A12* | Jul 2014 to Sep 2015 | 34.2 | 38.6 | 3.0 | 420 | | | | no | | | − | |
